# A Polymer-Gel Eye-Phantom for 3D Fluorescent Imaging of Millimetre Radiation Beams

**DOI:** 10.3390/polym10111195

**Published:** 2018-10-26

**Authors:** Leonard H. Luthjens, Tiantian Yao, John M. Warman

**Affiliations:** Delft University of Technology, Faculty of Applied Sciences, Department of Radiation Science and Technology, Section Radiation and Isotopes for Health, Mekelweg 15, 2629 JB Delft, The Netherlands; l.h.luthjens@tudelft.nl (L.H.L.); t.yao@tudelft.nl (T.Y.)

**Keywords:** radiotherapy eye-phantom, radio-fluorogenic gel, X-ray beam imaging, 3D radiation imaging, polymer gel dosimetry

## Abstract

We have filled a 24 mm diameter glass sphere with a transparent polymer-gel that is radio-fluorogenic, i.e., it becomes (permanently) fluorescent when irradiated, with an intensity proportional to the local dose deposited. The gel consists of >99.9% tertiary-butyl acrylate (TBA), pre-polymerized to ~15% conversion, and ~100 ppm maleimido-pyrene (MPy). Its dimensions and physical properties are close to those of the vitreous body of the human eye. We have irradiated the gel with a 3 mm diameter, 200 kVp X-ray beam with a dose rate of ~1 Gy/min. A three-dimensional (3D) (video) view of the beam within the gel has been constructed from tomographic images obtained by scanning the sample through a thin sheet of UV light. To minimize optical artefacts, the cell was immersed in a square tank containing a refractive-index-matching medium. The 20–80% penumbra of the beam was determined to be ~0.4 mm. This research was a preparatory investigation of the possibility of using this method to monitor the millimetre diameter proton pencil beams used in ocular radiotherapy.

## 1. Introduction

The treatment of ocular tumours is fraught with difficulties due to the proximity of important healthy tissues involved in sight and/or brain function. Radiotherapy has been an important method of treatment using either brachytherapy or external beam procedures [1,2,3,4]. The small dimension of the organ makes the use of highly collimated beams of millimetre dimensions imperative. With the recent proliferation of clinical proton beam sources [5], the incorporation of proton pencil beams in cancer protocols has become a worldwide reality [6,7,8]. Because of this, there is a current need for a medium capable of providing three-dimensional (3D) images with sub-millimetre spatial resolution of dose deposition in phantoms for confirmation of computer derived treatment protocols, equipment functioning, and for training clinical personnel [9].

A variety of methods involving 3D matrixes of one- and two-dimensional (2D) individual detectors are commercially available and presently used in the clinic. However, they are incapable of achieving the sub-millimetre spatial resolution required for small beam applications. For this, molecular media that undergo a measureable physico-chemical change on a microscopic scale when irradiated are required. Since the first suggestion in 1950 by Day and Stein [10] of using a quasi-rigid gel medium, several methods based on this basic formula have been developed and reviewed [11,12,13,14,15,16,17]. Unfortunately, none have become generally accepted in the radiotherapy clinic [18,19,20]. The reasons for this lack of adoption can be ascribed to a combination of: the complexity of the gel formulations, often involving up to five components, and the corresponding difficulty of universal reproducibility; the lack of off-the-shelf availability compared with gaseous (IC) or solid-state devices; the delay in post-irradiation data analysis; and, possibly, the cost.

In this report, we show how a radio-fluorogenic (RFG) polymer-gel [21,22] can be used to provide a 3D image of the track of a narrow (3 mm diameter) X-ray beam in a volume as small as the human eye. An RFG gel is a two-component mixture whose components are commercially available, inexpensive, and whose composition can be accurately determined by optical spectroscopy. The gel, which is tissue equivalent, becomes permanently fluorescent on irradiation with intensity proportional to the local dose deposited. The fluorescence can be tomographically scanned on-site immediately after irradiation and processed within minutes to yield 3D images of the radiation field [23]. The work reported here is a pilot study investigating the feasibility of applying RFG gel phantoms to the control of radiotherapy protocols and equipment for proton pencil beam treatment of eye tumours. It was driven by the recent construction of a proton radiotherapy clinic within the grounds of the authors’ institute. The capability of making 2D bulk images of proton pencil beams in an RFG gel was demonstrated previously by one of the authors using a high-energy physics particle accelerator [24].

## 2. Materials and Methods

### 2.1. RFG Gel Formation

The gel container used is a spherical borosilicate glass bulb 24 mm in outer diameter with a wall thickness of 1.7 mm; similar dimensions to those of the adult human eye. The bulb contains 5 mL of a transparent radiofluorogenic (RFG) gel with a gravimetric density of 0.91 kgL^−1^ and an electron density of 3.00 × 10^26^ L^−1^, values that are close to the 1.01 kgL^−1^ and 3.35 × 10^26^ L^−1^ of the vitreous body of the eye, and the 1.18 kgL^−1^ and 3.84 × 10^26^ L^−1^ of chemically-related PMMA (often denoted as “solid water”). The RFG gel consists of 15% pre-polymerised and inhibitor-free tertiary-butyl acrylate (TBA, Sigma Aldrich purum #01775, Sigma Aldrich company, Zwijndrecht, The Netherlands), to which is added approximately 100 ppm of the fluorogenic compound maleimido-pyrene (MPy, Sigma Aldrich P7908, Sigma Aldrich company, Zwijndrecht, The Netherlands).

**Figure 1:**
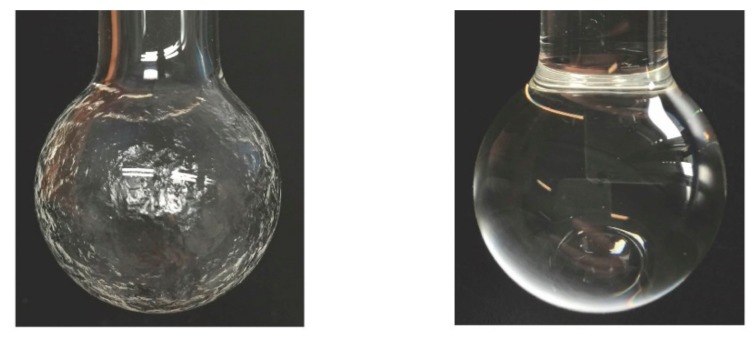
**Left**: The “eye-phantom” cell after radiation-induced polymerization of tertiary-butyl acrylate (TBA) to ~15% conversion and pumping-off the residual monomer leaving the polymer network. **Right**: The clear gel reformed on addition of a dilute solution of MPy in TBA and swelling of the polymer network.

The RFG gel preparation procedure has been described in full previously [21,22]. In summary, the gel is preformed in the cell by ^60^Co *γ*-irradiation (~15 Gy at ~1 Gy/min) of pure, de-aerated, and inhibitor-free TBA to ~15% monomer conversion. Excess monomer is then pumped off, leaving the polymer network deposited on the cell wall, as shown in Figure 1. The gel is reformed by adding a dilute solution of MPy in TBA equal to the volume of monomer pumped off and allowing time (~2 weeks) for reformation of the gel.

### 2.2. X-ray Beam Irradiation

The gel, contained in the eye-phantom cell, was irradiated with 200 kVp X-rays from a Philips MCN 321 X-ray tube. The conical beam was restricted by a brass collimator with a circular channel 3.0 mm in diameter and 30 mm long inserted in a 120 × 120 mm^2^ square, 27 mm thick Al/Pb/Al attenuator. The dose rate at the exit of the collimator, 460 mm from the source target, was ~1.0 Gy/min for a beam current of 15 mA. The cell was irradiated for 20 min resulting in a total incident dose of ~20 Gy. The basic irradiation set-up with optical rail, collimator and outline laser has been shown in a previous publication [23].

MD-V3 Gafchromic film (Lot Nr 02101602, Promis Electo-Optics B.V., Wijchen, The Netherlands) was used as a secondary, 2D dosimeter for monitoring the incident and exit beam dose rates and cross-sections. The dose sensitivity of the film was calibrated against an X-ray Exposure Meter (Vinten Instruments) that was routinely calibrated at the Netherlands Metrology Institute. The derivation of the dose from the measured intensity reflected by the scanned films has been described in full in reference [25]. The film was attached to the front and rear faces of a 24 mm square borosilicate glass cell placed at the same position as the 24 mm diameter eye-phantom cell. These control measurements were made with the square cell containing either air or an identical gel to that used in the eye-phantom.

### 2.3. Fluorescence Imaging

Fluorescence imaging was carried out using the ultraviolet (UV) slit scanning method previously reported [23]. This yields a series of tomographic images that can be used to generate a 3D image of the fluorescence within the gel. The sheet of UV light at 385 nm was 2 mm thick; the cell was transported in 1 mm steps through the sheet for a total distance of 30 mm. The cell was immersed in a glycerol bath contained in a 40 × 40 mm^2^ square borosilicate glass container with optically flat sides. This resulted in close matching of the refractive indexes of the surroundings (glycerol *n* = 1.47) with that of the borosilicate glass cell walls (*n* = 1.47) and the gel (*n* = 1.42). This diminished optical artefacts caused by lensing and reflections at dielectric interfaces.

## 3. Results and Discussion

### 3.1. Test Measurement with a Standard Fluorescent Solution

**Figure 2:**
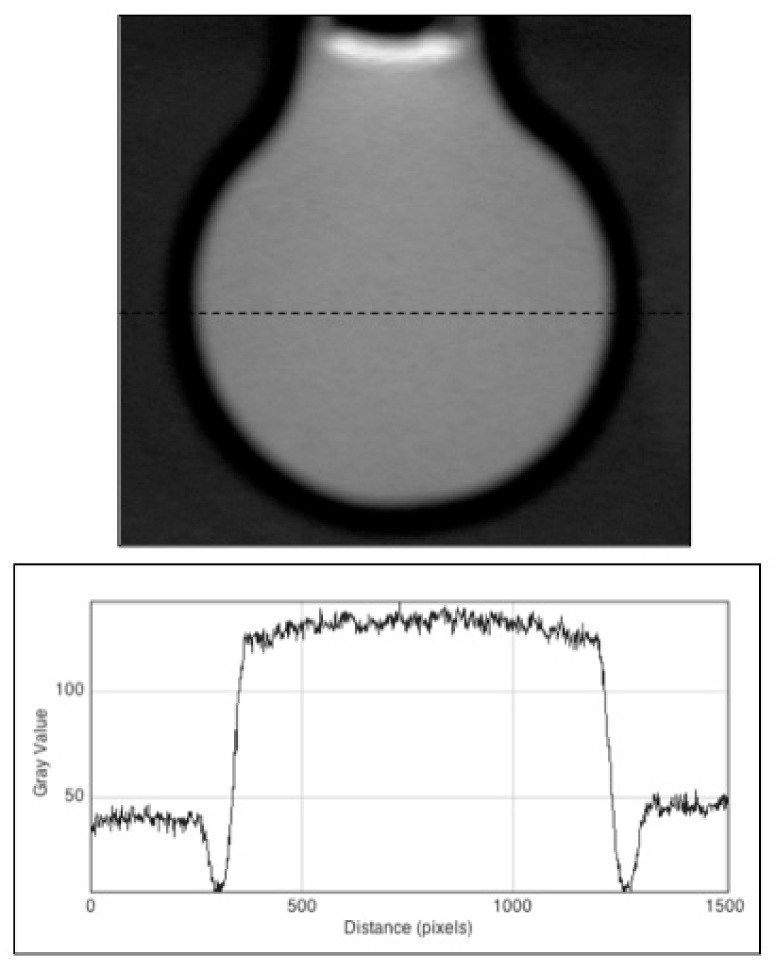
The fluorescence of a dilute solution of the fluorescence-standard diphenyl anthracene in the “eye-phantom” cell. The black “shadow” surrounding the cell, and corresponding intensity dips in the lower pixel profile, result from the non-fluorescent glass wall of the cell surrounded by the slightly fluorescent glycerol dielectric-matching bath. The position of the pixel profile scan is shown by the dashed line in the upper image. The pixel to pixel distance represents 0.023 mm in the gel.

In Figure 2 we show an image taken of a cell containing a micromolar concentration of the fluorescence-standard diphenyl anthracene (DPA) in cyclohexane. The image was taken with the UV excitation sheet positioned at the centre of the cell.

The pixel profile shown in Figure 2 displays a close to uniform fluorescence within the volume of the cell. The slight (~8%) upward curvature was most likely caused by a lensing effect due to the difference in refractive index between cyclohexane and glass (1.43 versus 1.47). The black “shadow” surrounding the liquid was due to the non-fluorescent glass cell wall. The slight increase in fluorescence outside the glass wall resulted from the fact that, in this measurement, the sample of glycerol used contained a fluorescent impurity. The presence of the impurity in the glycerol had a positive side to it, since it allowed us to determine the number of pixels per mm in the images from the accurately measureable outer diameter of the cell of 24.0 mm. The value determined was 44 pixels per mm, or 0.023 mm per pixel. This represents the ultimate practical limit to the spatial resolution of the measurements in the x/y plane. Additionally, thickness of the cell wall and the diameter of the gel could be accurately determined from the scan in Figure 2 to be 1.7 and 20.6 mm, respectively.

### 3.2. Radiochromic Film Measurements

**Figure 3:**
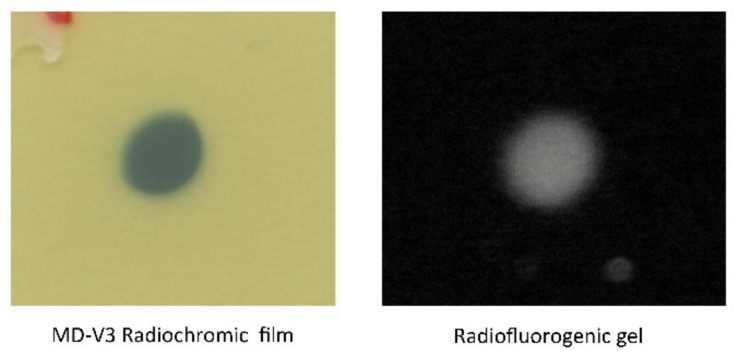
**Left**: The cross-sectional beam geometry as recorded using MD-V3 radiochromic film at the front face of the cell. **Right**: The fluorescent image as recorded in the radio-fluorogenic (RFG) gel looking along the propagation (z) axis of the beam.

An MD-V3 radiochromic film image of the X-ray beam incident on the cell is shown in Figure 3. As can be seen, the beam was slightly oval with a long axis 14% larger than the short axis. This phenomenon was attributed to the fact that the 3.0 mm (perfectly) circular collimator was smaller than the 4 mm dimension of the tungsten target of the source resulting in partial "pinhole imaging" of the X-ray emission from the target.

From a pixel profile line scan across the RC beam image in Figure 3 the 20–80% penumbra of the beam was found to be 0.41 and 0.54 mm for the incident and exit beams, respectively. The spatial resolution of the film images is 300 dpi or 0.085 mm/pixel. The much larger penumbra value was, therefore, attributed to edge-dispersion in the intensity of the X-ray beam.

The central optical absorption in the profile scan was used together with Equation (1) to determine the incident and exit dose rates. For a cell containing air, the values were 0.94 and 0.67 Gy/min; a decrease by 0.713. For a cell containing an RFG gel, the values were 0.89 and 0.47 Gy/min; a decrease by 0.528. The decrease in dose rate between the incident and exit films was due to the conical expansion of the beam from *z*_1_ (47.6 cm) to *z*_2_ (50.0 cm) and photon attenuation in the intervening media.

*D*’(z_2_)/*D*’(z_1_) = (*z*_1_/*z*_2_)^2^exp − {2[*M*ρδ]_glass_ + [*M*ρδ]_gel_} (1)

In Equation (1) *M*, ρ, and *δ* are the mass attenuation coefficient, the gravimetric density, and the length of the attenuating medium, respectively. For the air-containing cell, the attenuation by the cell contents was negligible; the overall decrease by a factor of 0.71 lead to a value of 2 [*M*ρδ]_glass_ = 0.244. Taking a borosilicate glass density of 2.3 g/cm^3^ and a wall thickness of 0.17 cm resulted in a mass attenuation coefficient for the cell wall of 0.312 cm^2^/g. From the dose rate decrease for the gel-containing cell we determined a value for (2 [*M*ρδ]_glass_ + [*M*ρδ]_gel_) of 0.540, or [*M*ρδ]_gel_ = 0.296 after subtracting the glass wall contribution determined above. Taking for the gel *δ* = 2.0 cm and *ρ* = 0.91 g/cm^3^ resulted in *M*_gel_ = 0.163 cm^2^/g, which is close to the value of 0.154 cm^2^/g previously found [26] and within the range of 0.21 to 0.13 cm^2^/g determined for 50 to 200 keV photons in PMMA, a compound of similar chemical composition [27].

### 3.3. Fluorescent Images of the Irradiated Gel

The irradiated cell was scanned in 1 mm steps with the UV sheet orthogonal to the propagation (*z*) axis of the X-ray beam. The resulting 30 tomographic images of the fluorescence were used to construct a 3D translucent image of the beam within the gel as described in reference [23,26]. This 3D representation can be viewed here in the form of a video either in full colour or in blue-pixel grey-scale: Video file links can be found in the Supplementary Materials (“Movie full color.MP4” and “Movie gray.MP4”).

**Figure 4:**
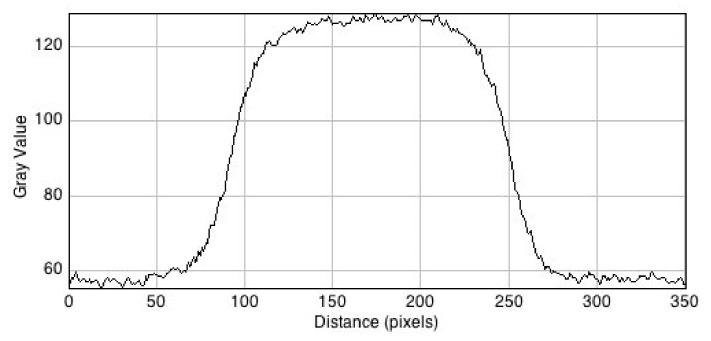
A pixel profile line scan across the center of the RFG gel image in Figure 3. The spatial resolution is 0.023 mm per pixel; the average 20–80% rise and fall value (the “penumbra”) is 0.44 mm.

An end-on view of the fluorescent image is shown in Figure 3 where it is compared with the incident beam cross-section found using the radiochromic film. The same oval form of the beam cross section is apparent in the RFG gel. A pixel profile scan across the beam is shown in Figure 4. From this, a penumbra value of 0.44 mm was found. This lies between the entrance and exit values of 0.41 and 0.54 mm found with the RC film. The spatial resolution of the profile for images taken in the x-y plane is 0.023 mm/pixel, which is considerably smaller than the penumbra value of 0.44 mm. As for the film measurements, the penumbra was ascribed to edge-dispersion in the X-ray beam, rather than spatial resolution of the measurements.

**Figure 5:**
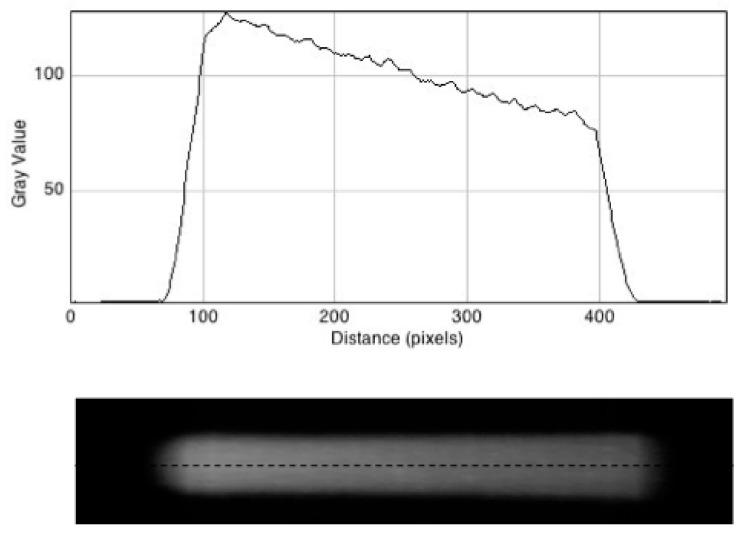
**Lower**: A still image of the reconstructed X-ray beam fluorescence side-on. **Upper**: A pixel intensity profile of the image taken along the dashed line shown.

In Figure 5 (lower) a side view (y/z plane) of the fluorescence reconstruction is shown. This displays a gradual decrease in intensity with increasing depth in the gel as expected.

The decrease is quantified in the pixel profile scan in Figure 5 (Upper) which decreases by a factor of 0.63 over the length of the gel. This is close to the value of 0.67, which would be expected for the decrease in dose rate across the gel based on the value of [*M*ρδ]_gel_ = 0.296, determined in Section 3.2, and a conical expansion by 10%.

Precise agreement might not be expected because of the superlinear dependence found for the fluorescence intensity on dose and the sublinear dependence found on dose rate [22]. These compensatory effects are the object of ongoing research into methods of making corrections to the experimental data required for dosimetry applications.

## 4. Conclusions

We have shown that it is possible to produce a three-dimensional, fluorescent image of the energy deposited by a 3 mm beam of high-energy radiation in a gel medium of physical properties and dimensions close to those of the vitreous body of the human eye. A 3D video image of the beam, with submillimetre spatial resolution, can be produced in-house within minutes of irradiation using a portable tomographic fluorescence-scanning apparatus with refractive index matching. We intend to apply the method to the study of energy deposition by proton pencil beams and to the control of radiotherapy protocols and equipment when the Holland Proton Therapy Clinic is eventually commissioned (www.hollandptc.nl/en/).

## Supplementary Materials

The following are available online at http://www.mdpi.com/2073-4360/10/11/1195/s1, Video S1: “Movie full color.MP4” and “Movie gray.MP4”.
